# Resource heterogeneity leads to unjust effort distribution in climate change mitigation

**DOI:** 10.1371/journal.pone.0204369

**Published:** 2018-10-31

**Authors:** Julian Vicens, Nereida Bueno-Guerra, Mario Gutiérrez-Roig, Carlos Gracia-Lázaro, Jesús Gómez-Gardeñes, Josep Perelló, Angel Sánchez, Yamir Moreno, Jordi Duch

**Affiliations:** 1 Departament d’Enginyeria Informàtica i Matemàtiques, Universitat Rovira i Virgili, Tarragona, Spain; 2 Departament de Física de la Matèria Condensada, Universitat de Barcelona, Barcelona, Spain; 3 Institute of Complex Systems UBICS, Universitat de Barcelona, Barcelona, Spain; 4 Department of Psychology, Comillas Pontifical University, Madrid, Spain; 5 Behavioural Science Group, Warwick Business School, University of Warwick, Coventry, United Kingdom; 6 Institute for Biocomputation and Physics of Complex Systems (BIFI), University of Zaragoza, Zaragoza, Spain; 7 Unidad Mixta Interdisciplinar de Comportamiento y Complejidad Social (UMICCS), UC3M-UV-UZ, Leganés, Spain; 8 Department of Condensed Matter Physics, University of Zaragoza, Zaragoza, Spain; 9 Grupo Interdisciplinar de Sistemas Complejos (GISC), Unidad de Matemática, Modelización y Ciencia Computacional, Universidad Carlos III de Madrid, Leganés, Spain; 10 Institute UC3M-BS of Financial Big Data, Universidad Carlos III de Madrid, Getafe, Spain; 11 Department of Theoretical Physics, University of Zaragoza, Zaragoza, Spain; 12 ISI Foundation, Turin, Italy; 13 Northwestern Institute on Complex Systems, Northwestern University, Evanston, Illinois, United States of America; Universidade de Lisboa, PORTUGAL

## Abstract

Climate change mitigation is a shared global challenge that involves collective action of a set of individuals with different tendencies to cooperation. However, we lack an understanding of the effect of resource inequality when diverse actors interact together towards a common goal. Here, we report the results of a collective-risk dilemma experiment in which groups of individuals were initially given either equal or unequal endowments. We found that the effort distribution was highly inequitable, with participants with fewer resources contributing significantly more to the public goods than the richer −sometimes twice as much. An unsupervised learning algorithm classified the subjects according to their individual behavior, finding the poorest participants within two “generous clusters” and the richest into a “greedy cluster”. Our results suggest that policies would benefit from educating about fairness and reinforcing climate justice actions addressed to vulnerable people instead of focusing on understanding generic or global climate consequences.

## Introduction

Mitigating anthropogenic climate change is a complex problem involving many heterogeneous actors with different agendas and socioeconomic conditions [1–5]. While climate change mitigation requires a collective action, it is not clear what are the effects of the inherent diversity of the agents involved in it [6]. There is the risk that the poor exploits the rich, i.e., that the largest beneficiaries of a common goods bear a disproportionately large burden in its production [7]. Conversely, the poor also have an incentive to contribute since they are risking as much as any other in the event of a catastrophic development.

Inequality between actors is a widespread situation related to climate change mitigation which occurs at all the socioeconomic scales, from large nations and corporations to citizens that share a given urban space. For instance, increasing green spaces and similar actions that improve urban environmental health are part of the Nationally Determined Contributions (NDCs) within the Paris Agreement (COP21) [8, 9]. However, these actions impact differently more and less affluent citizens and, in fact, such actions have also been questioned from the perspective of environmental justice [10]. This is the reason for the increasing interest in linking climate justice with socio-economic inequalities and in incorporating some behavioral aspects in city policy [11]. Therefore, understanding how to deal with climate change mitigation in an economically diverse world and in an environmentally fair manner is of special interest. This has become more timely and pressing with the withdrawal [12] of the second largest CO_2_ emitter [13] from COP21.

Resource heterogeneity and environmental justice can be very suitably framed within the collective-risk dilemma experimental setup introduced by Milinski *et al*. a decade ago [14]. In this framework, to be described in detail below, groups of people have to work together to reach a common goal by making contributions from an initial endowment. If the goal is reached, every individual receives the part of the money not contributed. If not, a catastrophe occurs with certain probability, and all participants lose all the money they had kept. While many experimental and theoretical studies have considered different aspects of climate change within this framework [15–32], the issue of heterogeneity has only been considered in three experiments framed in the climate change issue. Tavoni et al. [16] included inequality by separating the participants into two groups with different starting endowments, and found that the common goal was less likely to be reached. However, when they allowed participants to communicate their intentions, the probability of reaching the target goal increased again, similarly to conditional cooperation in public goods games [33]. On the other hand, Milinski *et al*. [17] observed that when they included rich and poor subjects, rich ones substituted for missing contributions by the poor provided intermediate climate targets, however, despite this increase in the contributions of the rich, the final target was reached less often than the intermediate target. Finally, Burton-Chellew and colleagues [21] proposed a game with four conditions varying the initial endowments and/or the risk of a catastrophic climate event. They found that inequality in both endowments and risk decreased cooperation, that is to say, selfishness emerged when rich were less at risk. However, some rich players were still reluctant to cooperate when they suffered the higher risk. It seems that climate change awareness could have mediated their responses, since it was found to be proportional with individual contributions. Outside of the climate change framing, studies on the role of heterogeneous endowments in public goods games are not conclusive, with different experiments leading to different results [34–36]. Therefore, new studies are needed to understand the influence of heterogeneity in threshold public goods games.

Here, we extend the knowledge on the effects of resource heterogeneity in collective dilemmas in two main directions. First, we include broader capital distributions, thus representing more closely the diversity in resource availability among the members of a given collective public goods game, e.g., between different countries worldwide or inhabitants in a given urban context. Second, and most importantly, we go beyond aggregate results to analyze the behavior of individuals by means of agnostic classification tools that allow us to identify differences between the behavior of subjects with the same resources. We complement this analysis with a questionnaire probing into the subjects’ knowledge of the climate change crisis and the influence of such knowledge in their actions. Therefore, we provide a much more complete picture of the influence of inequality that encompasses both the collective (reaching the common goal) and the individual (how different individuals behave under different circumstances) visions of the problem. Such a two-level approach is the best option in order to identify how agents react to resource heterogeneity and what actions must be taken to promote environmental justice. As we show below, our findings allow to hint directions for policy measures targeted to specific collectives.

## Materials and methods

The experiment was conducted following the lab-in-the-field experiment guidelines used in [37], which helped us recruiting participants from a general audience by using opportunistic recruitment, opposed to the typical samples of university undergraduate students. All participants in the experiment signed an informed consent to participate and no association was ever made between their real names and the results, in agreement with the Spanish Law for Personal Data Protection. This procedure was approved by the Ethics Committee of Universidad Carlos III de Madrid, and all methods were performed in accordance with the relevant guidelines and regulations. The experiment was conducted in different sessions in the DAU fair in Barcelona during two days (December 12-13, 2015) by means of the Citizen Social Lab platform developed ad hoc for this experiment [38]. The total number of games was 54, the number of participants in our experiment was 324 subjects, adding up to a total of 3240 game decisions collected. If some participant was non-responsive, the experimental platform took over and make the contribution randomly for her; in that case the data was labeled and the subject’s decisions were discarded in the analysis. The total number of subjects with uncorrupted decisions are 320 (134 women, 41.8%) leading to a total number of valid decisions of 3200. The age ranged from 11 to 73 years, 32.15 (13.04) on average (SD), there are no significant differences between the behaviour of minors (< 18) and adults at the level of individual contribution, nor between games in which there are minors and adults coexisting and in which there are only adults (S2 and S3 Tables). Almost half of the sample was graduated (48%), whereas the other half was equally distributed between different educational levels (i.e. professional training (16%), elementary (11%), middle (11%) and high school (12%). Most of the participants were naïve to social experiments (N = 279, 86%). Average (SD) earnings were 18.21 € (8.8) (S14 Fig) and the average (SD) duration of a game (considering only the time of decision making) was 82.21s (20.98), hence the average round time was 8.22s (S15 Fig).

Before starting the game, all the participants were shown a brief tutorial (S16 Fig) in the tablet in which the experiment was implemented. Researchers present in the experiment reviewed the instructions with them to guarantee they were understanding the basics of the experiment. Participants were reminded that they had to make a decision on each round on how much money they want to contribute to the common goal, but they were not instructed in any particular way nor with any particular goal in mind.

The subjects participated in groups of six players, each subject was assigned with an initial capital (20 € to 60 €), and the goal of the game was to contribute 120 € on a common fund between all of them. During the game the subjects had to contribute into the common fund between 0 € and 4 € during 10 rounds. At the end of each round all players saw the information of how much money has been contributed to the common fund, and they also saw the individual contributions of the six players in the previous round and the total amount contributed by each player up to this round (S16 Fig). If the goal was reached at the end of the game, all the participants kept the money that they had not contributed in the form of a gift card. The 120 € collected in the pot will be used once the research is published in organizing an event that includes an action against climate change (the action will consist in planting trees in a forest in Barcelona within the Day of the Tree event organized by an NGO [39]) and where there will be a dissemination of the current results. Otherwise, if the common fund did not reach 120 € at the end of the game, we did use the contribution to take any action against climate change and the participants only kept the remaining money with a probability of 10%.

### Statistical analysis

Numerical data representing payoff and contributions are expressed as mean±SE with a 95% CI. The sociodemographic (age and effect of minors), as well as earnings and time of decision-making are expressed as average (SD) and in the respective figures as standard error of the mean (95% CI). To control for potential sociodemographic data that could have an influence over the participant’s responses we used GLMM [40] with sex, age, education and the 2- and 3-way interaction between sex, age, education, followed by linear regression post test to establish the direction of the causality found at the educational level. Comparisons between average contribution and qualitative responses to question 3 (reaching the threshold) were conducted with non-parametric Chi Square. To explore how attitudes towards proportionality affected contributions, we sorted contributions according to the binomial response in question 7 (group 1 against group 2) and used non-parametric test for independent samples (Wilcoxon-Mann-Whitney test) for comparison. To mesure the differences among minors and adults in terms of contributions (both group and individual), we conducted Welch two sample t-test. Finally, to measure the robustness and stability of a given cluster we use consensus cluster metrics as item-consensus and cluster-consensus [41].

## Results

### The collective risk dilemma game

The original collective-risk dilemma [14] introduced groups of six people where each person receives an initial capital (40 €) and the common goal of the group is to collect 120 € that will be invested in mitigating climate change (by publishing an ad in a national newspaper). The game consists of 10 rounds, and at every round each subject decides how much she contributes to the common fund (0 €, 2 €, or 4 €). If the goal is reached at (or before) the end of the game, all participants keep the money that they have not contributed. Otherwise, the participants only keep the remaining money with a probability which in [14] was 90%, 50%, or 10% (equivalently, a climatic catastrophe occurred with probability 10%, 50%, or 90%). In addition, in this case no money goes to climate change mitigation. The main result was that most groups did not reach the goal, and even in the worst case scenario (catastrophe probability 90%) only about half of the groups avoided climate change. We reproduced the original dilemma with the worst case scenario and the exact same configuration as our baseline treatment, and we carried out sessions with wealth heterogeneity for comparison with the homogeneous one.

To introduce inequality, we distributed six different windfall starting capitals (20 €, 30 €, 40 €, 40 €, 50 €, and 60 €) randomly amongst the participants. In half of the games the participants could invest 0 €, 2 €, or 4 € per round as in the above setup, while in the other half of the games we allowed them more flexible choices, namely 0 €, 1 €, 2 €, 3 €, and 4 € per round (see S1 File Section 1.2). In all cases we informed the participants that in case they reached the goal of 120 €, the collected money would be used for a reforestation action by planting trees in a nearby park with the help of an NGO organisation [39]. Finally, after the experiment, we asked our subjects to answer a questionnaire (see S1 File Section 1.8) to have an individual assessment of climate change awareness and predisposition to collaborate in common actions that could be further correlated with the individual’s contributions.

### Equilibria and fair distribution

In our heterogeneous version of the collective-risk dilemma, there are very many Nash equilibria [42], which make claiming that particular behavior should be expected very difficult. Indeed, in the homogeneous case, the number of equilibria can be refined by imposing symmetry (meaning that all subjects, being equal, should choose the same contribution and, therefore, obtain the same payoff). Then, two equilibria are left, with individuals either contributing nothing or contributing exactly 20 €. Of course, these equilibria refer only to accumulated contribution along the game; considering the different sequences of investments in the 10 rounds recovers the multiplicity of equilibria and we will not consider them.

In our unequal setup we have 5 types of players (there are two endowed with 40 €) and the symmetry refinement no longer holds. Subjects contributing nothing is still a Nash equilibrium that leads to expected gains of 10% of every subject’s endowment. It is then easy to show that any combination of individual total investments that adds up to exactly 120 € such that every player makes more money than in the “contribute nothing” case is also an equilibrium. Therefore, there is not a clear theoretical prediction about what should happen in our heterogeneous version of the game.

**Fig 1:**
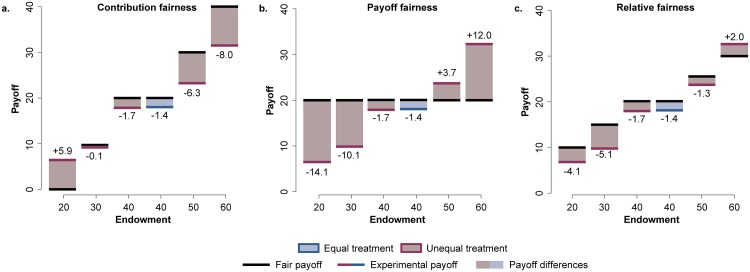
Comparison between the expected payoff of the three types of fairness versus the observed experimental payoff for each endowment in both equal and unequal treatments. **a. Contribution fairness**: in this case fairness is defined by equal contributions, where each participant is expected to contribute 1/6 of the common pot independently of their initial endowment. This definition yields particularly negative results for the most vulnerable participants, those that start with 20 €, who do not get any reward at all. We observe that in this case these participants prefer to keep part of their endowment so they have a chance to earn something. On the other hand, the rest of the groups contribute above the fair 20 € contribution. **b. Payoff fairness**: in this case fairness is defined by equal payoffs, where each participant is expected to receive the same payoff (20 €) independently of their initial endowment. This fairness forces the participants to end up with the same reward, that entails an increase of the experimental payoff differences. **c. Relative fairness**: in this third case we define fairness when all participants contribute half of their initial endowment, therefore, in this definition we expect to observe the same inequalities as before at the end of the game. Instead, we observe that in our experiments the inequality between the richest and the poorest increase.

While in the equal case in which the fair share behaviour was given by equal contribution and equal payoff (50% of the endowment in both cases), the asymmetric capital distribution in the unequal scenario requires three different concepts of fairness based on the same ideas. The first one refers to the equal payoff, “payoff fairness” (Fig 1b); the second one considers the criterion of equal contribution, “contribution fairness” (Fig 1a); and, finally we also analyze a “relative fairness” based on contributing half of the endowment (Fig 1c). The definitions of these quantifications of fairness are as follows: “payoff fairness” considers as fair behaviour the one in which each participant contributes an amount resulting in the same final payoff for all (i.e. 20 €). This means that participants receiving an endowment of 20 € should contribute 0 €, participants with an endowment of 30 € should contribute 10 €, and so on. “Contribution fairness” defines as fair an equal contribution (20 €) of each participant independently of the endowment. Finally, “relative fairness” considers as fair a 50% contribution of every subject’s initial endowment (i.e. 10 € for participants starting with 20 €, 15 € for participants starting with 30 €, and so on).

Among these possibilities, we decided to take the “relative fairness” as the main reference for the discussion of our results, which is the most representative of the inequalities generated by the participation in the game. If the participants contribute following “payoff fairness”, they start from a position of inequality (participants endowments from 20 € to 60 €) and once the game finishes their payoff is the same for all of them (20 €), breaking and unbalancing the initial inequalities due to the participation in the game. On the other hand, “contribution fairness” does not consider endowments at all, and therefore the initial inequality. However, in the case of “relative fairness”, after participating in the game the subjects maintain the same inequality distribution they started with. Therefore, we believe that this definition allows us to analyze the impact of the collective action in a more equitable and proportional way.

### Collective climate action

**Fig 2:**
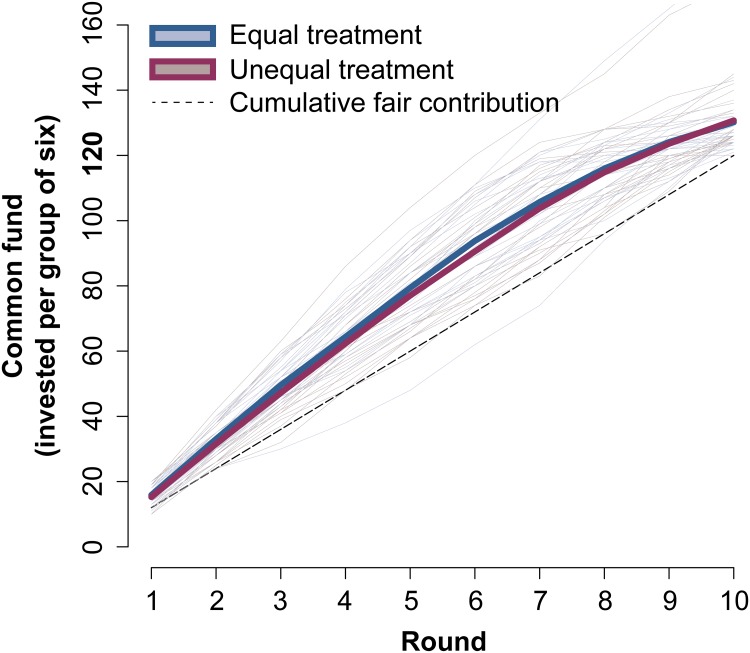
Average contribution to the common fund per round in the climate game for both, equal and unequal treatments. The equal treatment consists of 24 valid games in which all players are endowed with 40 €, and the unequal treatment consists of 26 valid games where the initial endowments of 20 €, 30 €, 40 €, 40 €, 50 €, and 60 € are randomly assigned to the players. In both treatments we observe that the accumulated contribution in all the rounds of all the games is almost always above the expected fair contribution per round (dotted line).

In all games played in our experiment, the participants reached the goal irrespective of the initial endowments being equal or unequal. In the former case, our result has to be compared with only 50% success rate for the groups in [14]. An increase in groups reaching the target has been observed in other similar studies carried out later [17]. The evolution of the games also differ from previous experiments (see Fig 2): In all the treatments in our experiment, the sum of money accumulated at the end of each round is always above the fair contribution (12 € per round), that is, participants contribute much more in the initial rounds, making the group reach the goal faster, and then they stop contributing at the end once the goal has been secured (S2 and S5 Figs). In contrast, the original results in [14] showed contributions below the fair one until the end of the game, and those groups that reached the goal did it by increasing their contributions in the last rounds.

### Effect of unequal capital distribution

**Fig 3:**
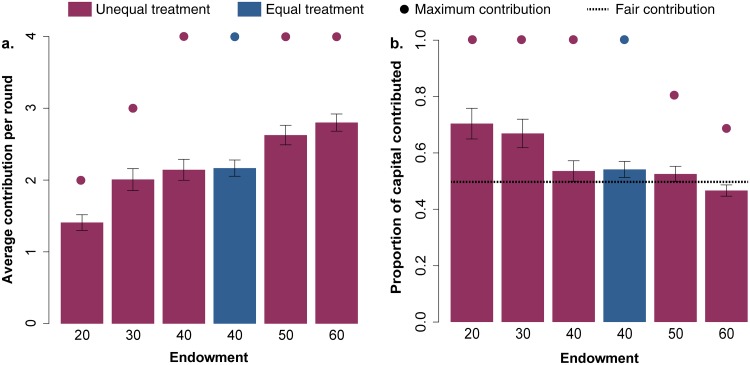
**a. Average (95% CI) capital contributed according to the participants’ endowments in both treatments equal and unequal**. Note that participants starting with 20 € and 30 € can only reach a maximum average contribution per round of 2 € and 3 € respectively. **b. Average (95% CI) proportion of capital contributed according to the participants’ endowments in both treatments equal and unequal**. Dotted line represents the fair contribution, which we have defined as contributing 50% of the initial capital. The effort to contribute is different depending on the endowments, so dots represent the maximum investment that each group can reach. Participants with endowments of 50 € and 60 € always keep a proportion of capital as savings even if they contribute the maximum amount of 4 € per round.

**Fig 4:**
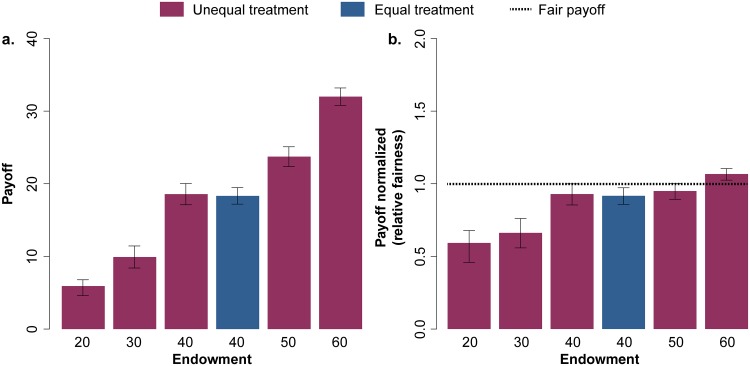
Average payoff for each of the initial endowment groups for the unequal (red) and equal (blue) treatments. Panel a shows the average payoff (95% CI) while panel b shows the values normalized following the definition of “relative fairness”, where each participant is expected to contribute half of their initial capital. We observe that those who have more resources get a higher normalized payoff, and in the 60 € group it is even above the expected fair. Instead, the most vulnerable (20 € and 30 €) get a much lower payoff than the fair. There are significant differences between them and the rest of participants with higher endowments (S4 and S5 Tables).

Fig 3 presents the average amount of capital contributed as a function of the initial capital of the participants. We observe that, in terms of absolute contribution, the subjects with high endowments, 50 € and 60 €, are the ones that contribute the most, 2.6 ± 0.13 € and 2.8 ± 0.12 € per round respectively (mean±SE). Participants with low-endowments, 20 € and 30 €, contribute the least,1.4 ± 0.11 € and 2 ± 0.15 € respectively. However, this comparison is not the best for interpreting the obtained results, since the initial capital of the poorest players only allows them to contribute a maximum of 2 € per round (earning 0 at the end). Therefore, the comparison makes more sense in terms of the percentage of capital contributed relative to their total capital, which in turn allows to discuss the results using the “relative fairness” as reference (Fig 4). Strikingly, we observe that the most affluent (endowments of 60 €) are the ones that contribute proportionally less, with around 46.6% of their initial capital, while the poorest (starting with 20 €) contribute around 71.4% of their initial capital which shows their vulnerability when facing the collective risk dilemma. Fig 3 shows very clearly the stark contrast between the two visions. To put this result further in context, we notice that the maximum contribution from participants with an endowment of 20 € (2 € per round) implies an effort of 2 times the fair contribution, whereas for participants with an endowment of 60 €, the effort of contributing 2 € per round is 0.66 times the fair contribution. Therefore, in that case, contributing 2 € have unequal impacts in the capital of subjects: the poorest participants, contributing 1.4 € on average, make a larger effort than wealthy participants contributing 2.8 €, which is even below their fair share of the threshold.

### Individual behaviors

Once we have looked at the average evolution of the different groups, we focus on the individual behavior and study whether participants with the same starting capital behave similarly. We characterize the set of decisions taken by each participant with a vector, grouping decisions by the capital on the common fund at the beginning of the round (see S1 File Section 1.3 for a more detailed explanation). This is motivated by the intuition that subjects choose their contributions as a function of their endowment but also taking into account the current situation and whether the goal is closer or farther. In turn, we can monitor how the contributions differ depending on the stage of the game where they are (S6 and S7 Figs). Next, in order to detect, identify and characterize different types of behavioral patterns, we use an unsupervised learning algorithm, namely Ward’s hierarchical clustering method [43, 44] with squared Euclidean distances. Additionally, we used a consensus clustering to look for the optimal subdivision of our data into groups as well as for the robustness of each group (see S1 File Section 1.4). This allowed us to find the groups that better fit the collected data as well as a much more stable solution [41].

**Fig 5:**
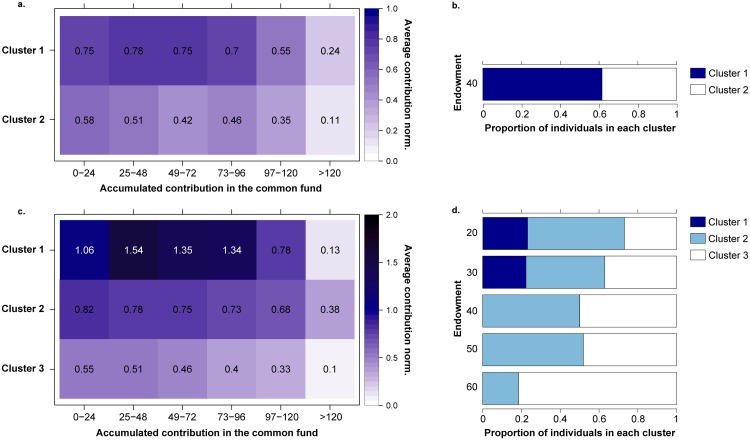
**a. Behavioral patterns in the equal treatment based on average contribution during the evolution of the game**. The value in each cell represents the average contribution normalized by the initial capital per round (i.e: 2 for participants starting with 20 €, 3 for participants starting with 30 €, and so on; 0.5 is the fair contribution) in a given stage of the game (depending on the accumulated contribution at that stage). **b. Proportion of individuals in the equal treatment groups**. Cluster 1 is formed by generous subjects (61%) with average contribution above the fair while cluster 2 is formed by subjects (39%) that contribute around and below the fair contribution. Unequal treatment. **c. Behavioral patterns in the unequal treatment based on average contribution during the evolution of the game**. The value in each cell represents the average contribution normalized by the initial capital per round in a given stage of the game (depending on the accumulated contribution at that stage). Cluster 1 consists of hyper-generous individuals (7.45%) that contribute very much above fair, cluster 2 is formed by generous individuals (43.48%) with average contribution above fair, and cluster 3 is formed by individuals (49.07%) that contribute around and below the fair contribution. **d. Proportion of individuals in the unequal treatment groups**. Distribution of the different types among the participants as a function of their initial endowment.

In Fig 5a and 5b we present the results of the clustering of the participants of the homogeneous treatment, with equal capital distribution. In this scenario the optimal number of groups identified is two. Looking at the results of the clustering we immediately identify two different types of behaviors, a group of generous participants (cluster 1) that contribute above the fair contribution, and a group of more greedy participants that contribute around the fair contribution at the beginning of the game and decrease their contribution as they approach the end of the game (but before reaching the goal).

The results for the unequal treatment in Fig 5c and 5d show that the optimal division of the participants is into three groups, being clusters 2 and 3 those gathering the majority of subjects (92.55%), similarly to the equal treatment. Nevertheless, in this case emerge a minority group (7.45%) of hyper-generous individuals (cluster 1) that contributes far beyond what we are considering fair. And, again, in cluster 2 the subjects contribute on average more than the fair contribution, whereas in cluster 3 the average contributions are around the fair value at the beginning, but as the game approaches the end they decrease below the fair amount. We also observe in this treatment that subjects of the hyper-generous group increase their contributions significantly after the initial rounds, while the rest of the groups maintain or decrease their contribution as the game advances.

In this latter framework, participants have different initial endowments. Thus, it is interesting to check how these subjects are distributed in each of the three groups based on their relative contribution. Fig 5d shows that subjects with fewer resources (20 €-30 €) than the rest are concentrated in the generous clusters (1 and 2). In fact, the cluster 1 (the hyper-generous group) is formed exclusively by subjects with low endowments. On the other hand, the third cluster is mainly composed by subjects with mid and high endowments. This means that the majority of low endowed participants, 73.07% (20 €) and 62.96% (30 €) contributed above the fair threshold, different from the subjects with high endowments, where only 51.85% (50 €) and 18.51% (60 €) contributed a fair amount. Interestingly, the comparison of Fig 5b and 5d shows that subjects with mid endowments (40 €-50 €) distributed among the two clusters not very differently from the equal treatment. Therefore, the richest participants were those who diverged from that distribution.

### Effect of awareness about climate change

All groups reached the goal. Although climate awareness has proven to have an effect on individual responses [21], the majority of our sample (N = 294, 91.3%) failed basic questions about climate change concepts, in a questionnaire that included basic questions about the greenhouse effect, carbon footprint, or the Kyoto Protocol (see S1 File Section 1.8). We stress that this is so even if the experiment was done the week following the COP21 summit in Paris, which had led us to expect much more familiarity of our subjects with climate change. This result allows us to exclude that more literacy and more public outreach efforts on climate change are the reasons for the participants reaching the goal in all cases.

### Effect of socio-demographics and beliefs

No significant differences were observed in terms of age or gender of the participants. However, educational level was a factor that significantly affected the average contribution (GLMM, *χ*^2^ = 3.811, df = 1, p = .006). Subjects with lower education level were predicted to make higher contributions in equal conditions (F(1,156) = 7.219, p<.05). In addition, before starting the game a third part of the sample (N = 112, 34.8%) expected to arrive to the common goal. Harboring this previous expectation did not have an influence over their average contribution (*χ*^2^ = 6.005, df = 3, p = .111).

Importantly, we detect some inconsistencies between belief and behavior. The majority of the sample (around 87%) claimed that their contributions should not depend on the co-participant’s contribution, but the existence of different clusters shows that they did take the accumulated capital into account to decide their next contribution. In addition, more than half of the participants (N = 214, 66%) defended the idea of relative fairness (e.g. agreed with the statement “Contributions should be proportional to the initial capital so that players with more capital should contribute more to the pool”). In contrast, those who firmly agreed with proportionality in the unequal conditions contributed less than those who did not strongly support that (Z = -2.653, *p* < .05), especially when their initial endowment was high. For example, the participants with a starting capital of 60 € that adhered to “proportionate contributions” contributed an average of 2.6 € per round, whereas those with the same initial endowment who did not firmly claim that contributed 3.05 € per round.

### Effect of generosity on emergence of inequality

Finally, we measured what the impact of this selfless behaviour among the poorest participants on the global inequality was. Gini Coeficcient, apart from a standard way to calculate inequality levels in economics, is completely appropriate here since general behaviour consisting on contributing the fair share would leave the value of Gini Coefficient constant (S13 Fig). Thus, in the equal treatment the Gini Coefficient increases from 0 (initial distribution) to 0.1806 at the end of the experiment, whereas in the unequal treatment increases from 0.1812 to 0.3483.

## Discussion

Our experimental results allow us to conclude that inequalities may lead to unexpected problems in climate change mitigation, mostly related to environmental justice. Interestingly, knowledge about climate change facts did not play a relevant role in the average contributions to the common fund. This might indicate that policies mainly addressed to increase climate change awareness might not be the most efficient solution to foster cooperation, and suggests that emphasizing a correct interpretation of the perceived effects might be more useful in this regard.

Even if all groups avoided the climatic catastrophe, our work shows that other potentially serious issues may arise in the process. A particularly important one is that disadvantaged individuals are contributing much more than a fair share of the mitigation, and that the richest ones are contributing less. It is telling that all hyper-generous behavior is observed in the two poorest types of individuals, while a large majority of those endowed with the largest amount behaved selfishly (irrespective of what they claim to believe about fair contributions, as we have seen). It thus appears that, contrary to the expectations of the poor exploiting the rich in a public goods context, here we found the opposite situation. This result has also been pointed out in other studies with even higher unequality [45], where they also observe a selfish behavior of the richest participants.

At this point, it is important to note that these experiments were done in a short period of time and therefore they do not inform about long-term behavior. It would be possible that the fact that the poor contributed more for a long time might eventually lead them to stop doing so, thus jeopardizing the collective goal. The decisions we observed in the experiment did not allow us to learn about the behavior of the richest players in that case, i.e., whether or not they would jump in to solve the problem (although the results in [17] suggest that this could be the case).

Finally, it is important to discuss the different behaviors observed in the collectives we have worked with. Education does not help here: less educated-less favored participants contributed more to the collective goal than more educated-more favored ones. This could indicate that there is an underlying cultural assumption of sacrifice of the most disadvantaged people (related to their vulnerability): in a situation where the poorest are the ones who will face the worst consequences, more advantaged participants may feel inclined to contribute less to solving the problem. Particularly alarming is the fact that, in the group of the richest participants, about 80% behaved in a selfish manner. As this is the group that had the largest means to help to mitigate climate change, their fault to contribute may jeopardize the whole society, which calls for specific actions to work with this segment of the population while providing additional policies to protect more disadvantaged groups or collectives.

To suggest new ways to initiate a collective climate action according to our findings, firstly it is worth noting some differences of our study with a real world situation. For example, similar to [21], our participants gathered information about their co-participants’ responses whereas particular information from others (i.e. foreign countries or other communities sharing space in a city) may not be available, reliable, or complete in the real world. Therefore, to foster cooperation, future policies may benefit from making data and contributions public and transparent. Moreover, our experimental subjects held unambiguous responsibility over their actions whereas climate change is a global problem with diffuse shared duties. In this sense we have proven that a good general education is not the remedy to avoid inequality in contribution, but promoting collective rather than parochial attitudes, which has proven to be one factor underlying cooperation [45], may be a better solution to make individuals assume their responsibilities. Finally, we used an economic paradigm to establish a concrete threshold to be reached so that our subjects received the economic consequences directly and immediately once the game was over, however the Nature’s threshold is more uncertain [46] and consequences can spread out over generations. Monetizing Nature by establishing concrete thresholds to be reached in a particular time period and rewarding the population if they enact some actions (i.e. return taxes if substituting diesel oil cars with electric vehicles or if groups of neighbors install and maintain a rooftop garden on their own buildings) may work to address people towards a general cooperation.

## Supporting information

S1 Fig Sociodemographic. Distribution of subjects in the experiment by age and gender.
(PDF)

S2 Fig Investment choices at the beginning and end of the game. Density of investment selections, mean and standard error of the mean (95% CI), in the first five rounds and the last five rounds. a. Equal treatment and investment options of 0-2-4. b. Unequal treatment and investment options of 0-2-4. c. Equal treatment and investment options of 0-1-2-3-4. d. Unequal treatment and investment options of 0-1-2-3-4.
(PDF)

S3 Fig Proportion of savings depending on the investment options and the endowments. a. Proportion of savings, mean and standard error of the mean (95% CI), at the end of the game per endowment and investment treatment. b. Differences of remaining capital −savings (S)− between the treatment 0-1-2-3-4 and 0-2-4 per endowment (*S*_01234_ − *S*_024_ per endowment in each round).
(PDF)

S4 Fig Average individual investment and standard error of the mean (95% CI) by treatment over the game evolution. In both equal treatment and unequal treatment, participants’s contribution decreases along the game. The differences between the two treatments are not statistically significants (MWU Two-Sided, U: 50.0, P: 0.97).
(PDF)

S5 Fig Number of games in which the goal has been achieved in a particular round. The average (SD) round is 8.83 (1.07).
(PDF)

S6 Fig Average individual investment and standard error of the mean (95% CI) by treatment over the game evolution (bin = 12). Decisions are grouped according to the total capital invested on the common fund at the start of the round. In both equal treatment and unequal treatment participants contribute above the fair contribution in the first part of the game and decrease when they are close to reach the target. We can observe three different regions on the game evolution: first, from 0 €-30 € participants are more erratic and at the same time contribute more to the average value. Second, from 30 € to 90 € approximately there is a stable contribution slightly above the ideal average contribution. And third, after 90 € and until the goal is reached participants decrease substantially their final contribution.
(PDF)

S7 Fig Distributions of normalized contributions in the three phases of the game. The mean (SD) in each phase, based on the accumulated capital in the common fund, is: common fund from 0 to 30 €: 0.67 (0.33), common fund from 31€ to 96 €: 0.62 (0.37), and common fund from 97 € to 120 €: 0.39 (0.38).
(PDF)

S8 Fig Ideal “pure” strategies based on our experiment design. (PDF)

S9 Fig Equal treatment clustering. (Top-Left) Optimal number of clusters. (Top-Right) Cluster consensus ratio. (Bottom) Item consensus ratio.
(PDF)

S10 Fig Equal treatment distributions. (Left) Distribution of subjects in clusters based on their average contribution per round. (Right) Cumulative distribution function based on their average contribution per round.
(PDF)

S11 Fig Unequal treatment clustering. (Top-Left) Optimal number of clusters. (Top-Right) Cluster consensus ratio. (Bottom) Item consensus ratio.
(PDF)

S12 Fig Unequal treatment distributions. (Top) Cumulative distribution function based on their average contribution per round. (Bottom) Distribution of subjects in clusters based on their average contribution per round.
(PDF)

S13 Fig Distribution of capitals in different scenarios of the game for the equal treatment (first row in light blue) and the unequal treatment (bottom row in red). First column displays the corresponding distribution of endowments at the beginning of the game. Second column pictures the hypothetical final distribution if all the participants contributed fairly, i.e. with half of their endowments. Finally in the third column, the observed final distribution of capitals is shown. The Gini coefficient is indicated in every case.
(PDF)

S14 Fig Earnings. Average earnings and standard error of the mean (95% CI) regarding treatment and endowments.
(PDF)

S15 Fig Decision making times. (Left) Duration of a game, mean and standard error of the mean (95% CI), per treatment. (Right) Evolution of decision making times over round.
(PDF)

S16 Fig Screenshots of the tutorial shown before the experiment. Images of the character created by Mensula Studio are licensed under CC BY 4.0.
(PDF)

S1 Table Example of user’s contribution normalization and binning in a particular game. (PDF)

S2 Table Cohort analysis of game contributions in games with and without minors. (PDF)

S3 Table Cohort analysis of contribution per endowment in groups of minors and adults. (PDF)

S4 Table Payoff and payoff normalized by relative fairness. (PDF)

S5 Table Pairwise comparation of payoff normalized by relative fairness. (PDF)

S1 File Supporting information file. In the S1 File (PDF) we present further details about the experiment, statistical tests, clustering analysis and additional information.
(PDF)
