# Supplementary figures and images for: Resource heterogeneity leads to unjust effort distribution in climate change mitigation
Source: PLoS One. 2018 Oct 31;13(10):e0204369. doi: 10.1371/journal.pone.0204369 (PMC6209147; doi:10.1371/journal.pone.0204369)

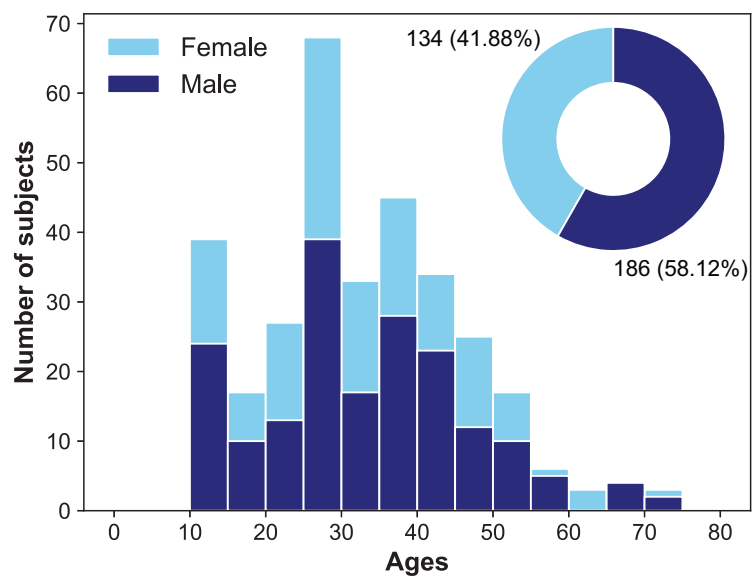

**Fig S1: Sociodemographic** Distribution of subjects in the experiment by age and gender.

Supplement: S1 Fig — Distribution of subjects in the experiment by age and gender. (PDF) [file pone.0204369.s001.pdf]

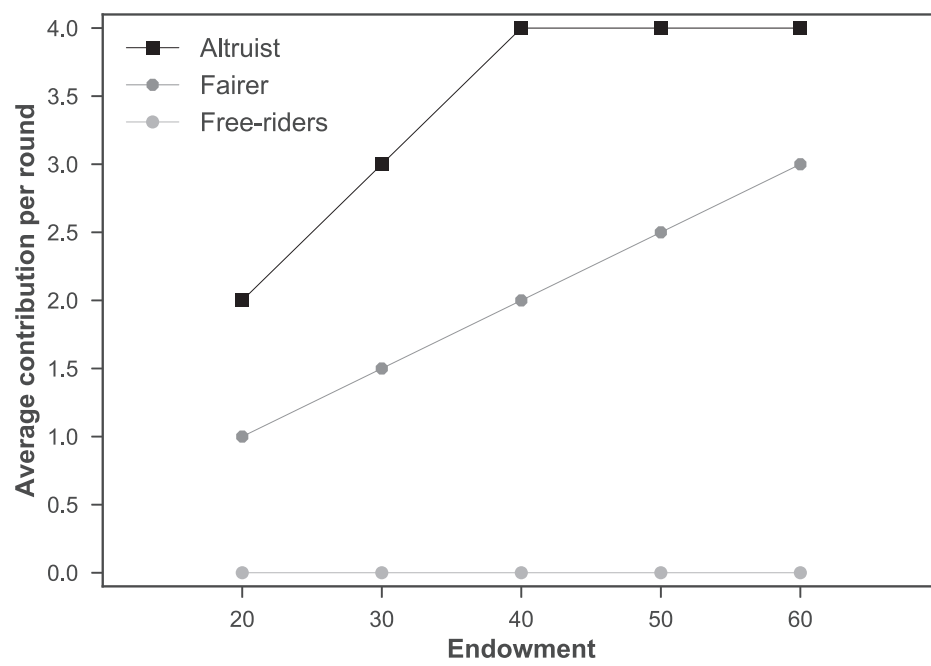

Fig S8: Ideal "pure" strategies based on our experiment design.

Supplement: S8 Fig — (PDF) [file pone.0204369.s008.pdf]
