# Supplementary material for: Resource heterogeneity leads to unjust effort distribution in climate change mitigation
Source: PLoS One. 2018 Oct 31;13(10):e0204369. doi: 10.1371/journal.pone.0204369 (PMC6209147; doi:10.1371/journal.pone.0204369)
Supplement: S2 Fig — Density of investment selections, mean and standard error of the mean (95% CI), in the first five rounds and the last five rounds. a. Equal treatment and investment options of 0-2-4. b. Unequal treatment and investment options of 0-2-4. c. Equal treatment and investment options of 0-1-2-3-4. d. Unequal treatment and investment options of 0-1-2-3-4. (PDF) [file pone.0204369.s002.pdf]

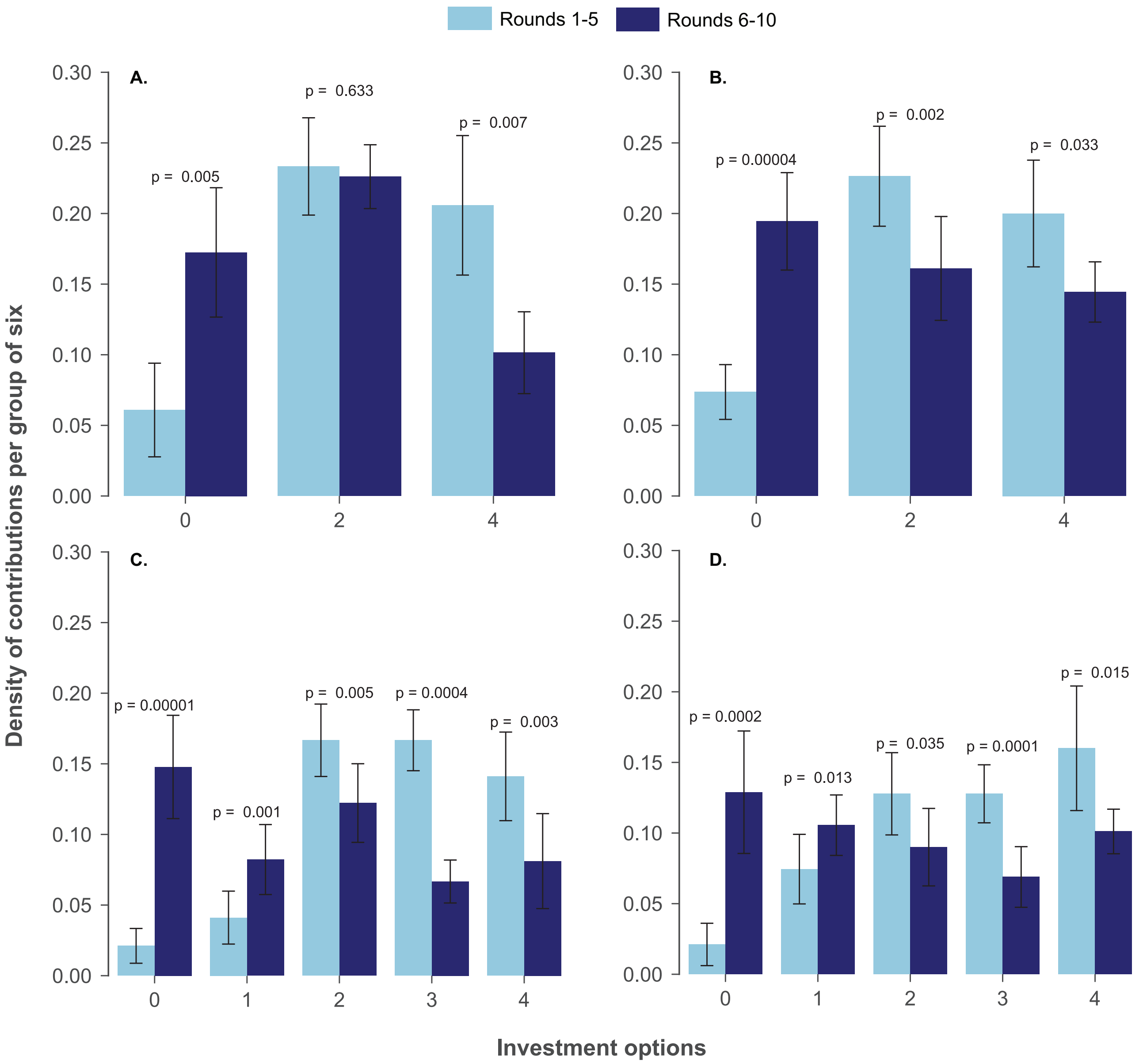

Fig S2: Investment choices at the beginning and end of the game. Density of investment selections, mean and standard error of the mean (95% CI), in the first five rounds and the last five rounds. a. Equal treatment and investment options of 0-2-4. b. Unequal treatment and investment options of 0-2-4. c. Equal treatment and investment options of 0-1-2-3-4. d. Unequal treatment and investment options of 0-1-2-3-4.
