# Supplementary material for: Resource heterogeneity leads to unjust effort distribution in climate change mitigation
Source: PLoS One. 2018 Oct 31;13(10):e0204369. doi: 10.1371/journal.pone.0204369 (PMC6209147; doi:10.1371/journal.pone.0204369)
Supplement: S3 Fig — a. Proportion of savings, mean and standard error of the mean (95% CI), at the end of the game per endowment and investment treatment. b. Differences of remaining capital −savings (S)− between the treatment 0-1-2-3-4 and 0-2-4 per endowment (S01234 − S024 per endowment in each round). (PDF) [file pone.0204369.s003.pdf]

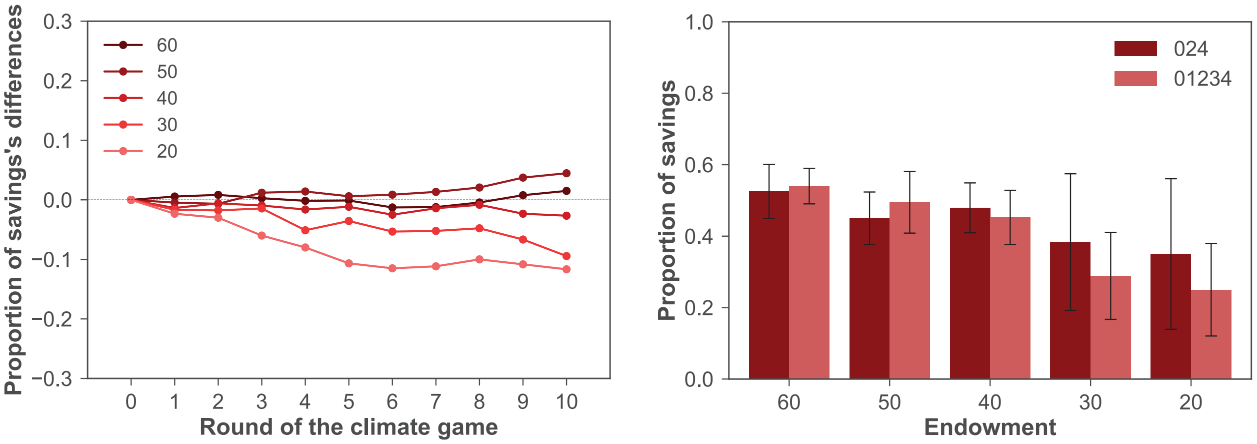

Fig S3: Proportion of savings depending on the investment options and the endowments. (Left) Proportion of savings, mean and standard error of the mean (95% CI), at the end of the game per endowment and investment treatment. (Right) Differences of remaining capital –savings (S)– between the treatment 0-1-2-3-4 and 0-2-4 per endowment ($S_{01234} - S_{024}$ per endowment in each round).
