# Supplementary material for: Resource heterogeneity leads to unjust effort distribution in climate change mitigation
Source: PLoS One. 2018 Oct 31;13(10):e0204369. doi: 10.1371/journal.pone.0204369 (PMC6209147; doi:10.1371/journal.pone.0204369)
Supplement: S4 Fig — In both equal treatment and unequal treatment, participants’s contribution decreases along the game. The differences between the two treatments are not statistically significants (MWU Two-Sided, U: 50.0, P: 0.97). (PDF) [file pone.0204369.s004.pdf]

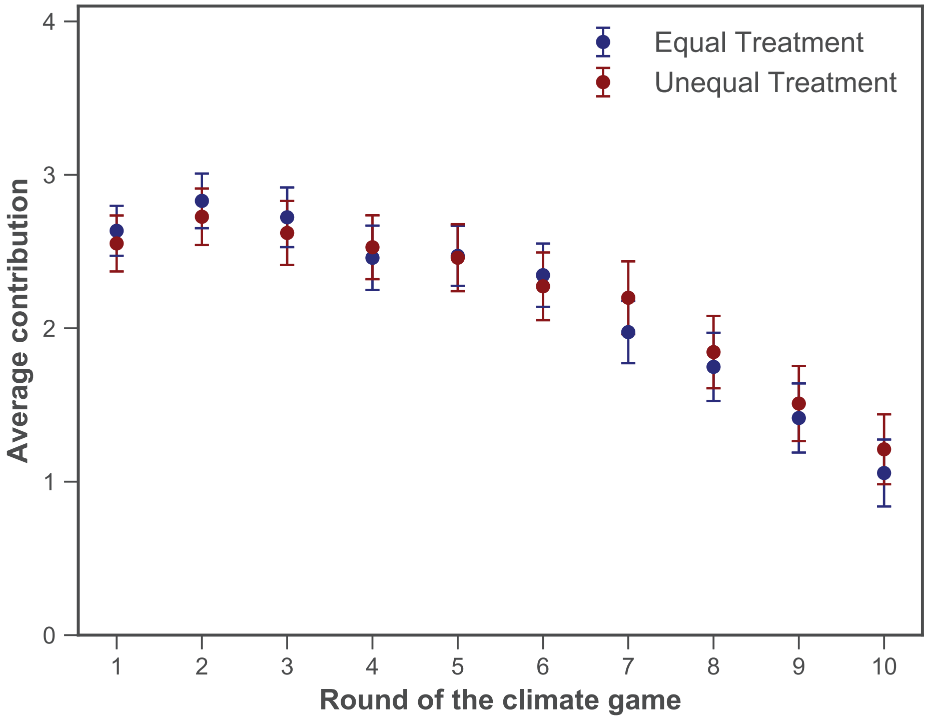

Fig S4: Average individual investment and standard error of the mean (95% CI) by treatment over the game evolution. In both equal treatment and unequal treatment, participants's contribution decreases along the game. The differences between the two treatments are not statistically significants (MWU Two-Sided, U: 50.0, P: 0.97).
