# Supplementary material for: Resource heterogeneity leads to unjust effort distribution in climate change mitigation
Source: PLoS One. 2018 Oct 31;13(10):e0204369. doi: 10.1371/journal.pone.0204369 (PMC6209147; doi:10.1371/journal.pone.0204369)
Supplement: S5 Fig — The average (SD) round is 8.83 (1.07). (PDF) [file pone.0204369.s005.pdf]

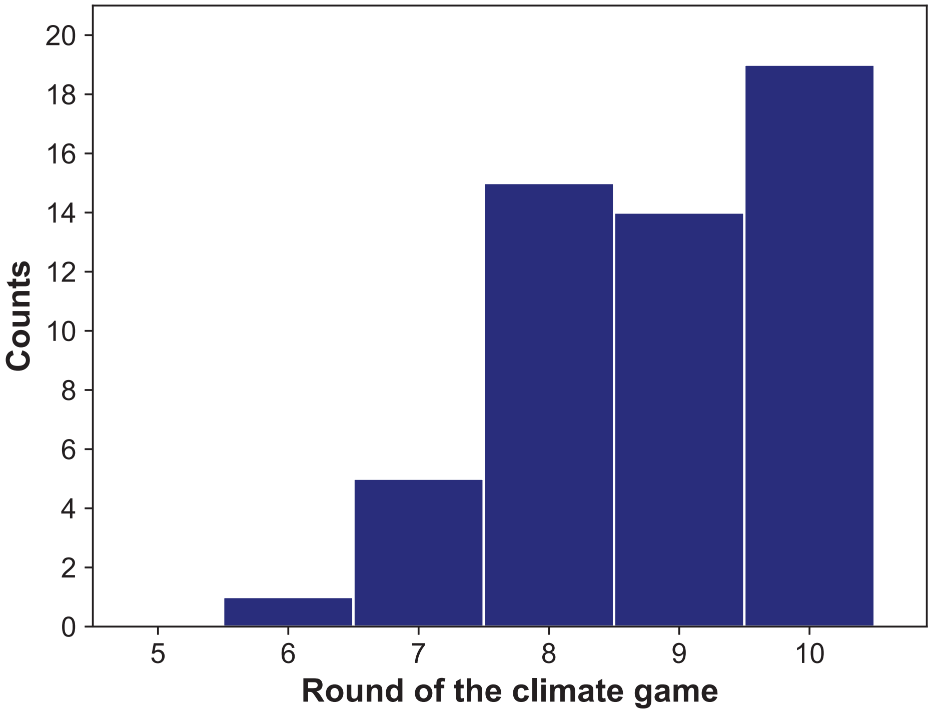

Fig S5: Number of games in which the goal has been achieved in a particular round. The average (SD) round is 8.83 (1.07).
