# Supplementary material for: Resource heterogeneity leads to unjust effort distribution in climate change mitigation
Source: PLoS One. 2018 Oct 31;13(10):e0204369. doi: 10.1371/journal.pone.0204369 (PMC6209147; doi:10.1371/journal.pone.0204369)
Supplement: S6 Fig — Decisions are grouped according to the total capital invested on the common fund at the start of the round. In both equal treatment and unequal treatment participants contribute above the fair contribution in the first part of the game and decrease when they are close to reach the target. We can observe three different regions on the game evolution: first, from 0 €-30 € participants are more erratic and at the same time contribute more to the average value. Second, from 30 € to 90 € approximately there is a stable contribution slightly above the ideal average contribution. And third, after 90 € and until the goal is reached participants decrease substantially their final contribution. (PDF) [file pone.0204369.s006.pdf]

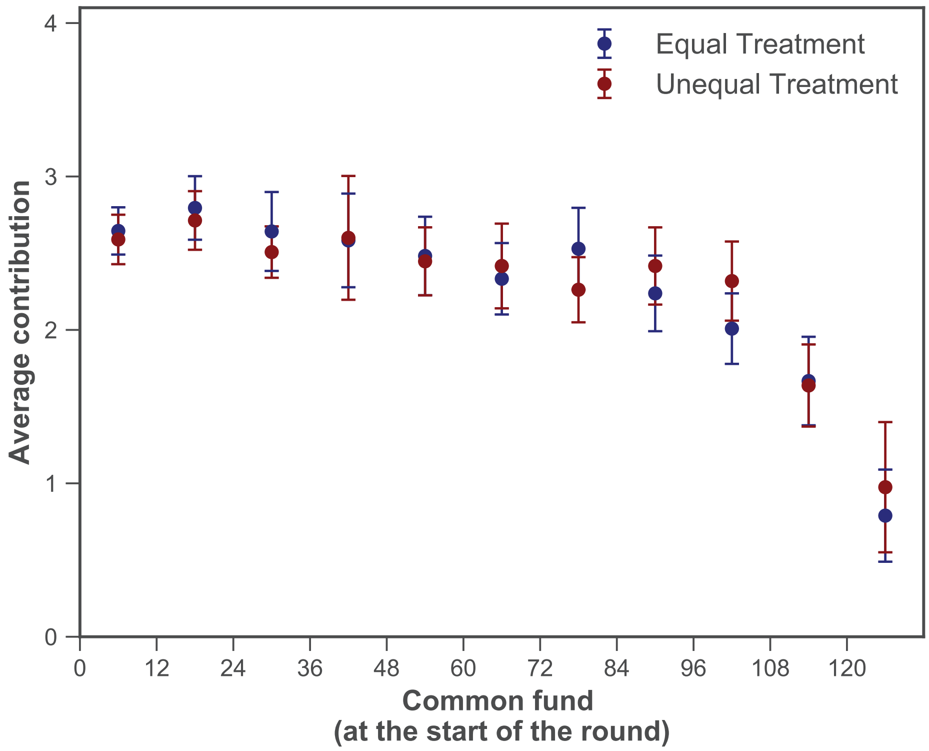

Fig S6: Average individual investment and standard error of the mean (95% CI) by treatment over the game evolution (bin=12). Decisions are grouped according to the total capital invested on the common fund at the start of the round. In both equal treatment and unequal treatment participants contribute above the fair contribution in the first part of the game and decrease when they are close to reach the target. We can observe three different regions on the game evolution: first, from 0 €-30 € participants are more erratic and at the same time contribute more to the average value. Second, from 30 € to 90 € approximately there is a stable contribution slightly above the ideal average contribution. And third, after 90 € and until the goal is reached participants decrease substantially their final contribution.
