# Supplementary material for: Resource heterogeneity leads to unjust effort distribution in climate change mitigation
Source: PLoS One. 2018 Oct 31;13(10):e0204369. doi: 10.1371/journal.pone.0204369 (PMC6209147; doi:10.1371/journal.pone.0204369)
Supplement: S7 Fig — The mean (SD) in each phase, based on the accumulated capital in the common fund, is: common fund from 0 to 30 €: 0.67 (0.33), common fund from 31€ to 96 €: 0.62 (0.37), and common fund from 97 € to 120 €: 0.39 (0.38). (PDF) [file pone.0204369.s007.pdf]

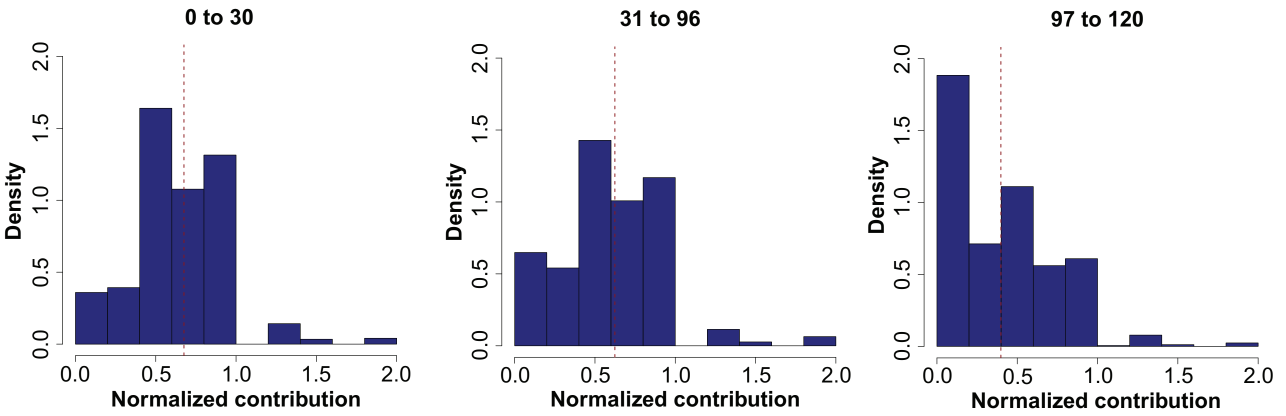

Fig S7: Distributions of normalized contributions in the three phases of the game. The mean (SD) in each phase, based on the accumulated capital in the common fund, is: common fund from 0 € to 30 €: 0.67 (0.33), common fund from 31 € to 96 €: 0.62 (0.37), and common fund from 97 € to 120 €: 0.39 (0.38).
