# Supplementary material for: Resource heterogeneity leads to unjust effort distribution in climate change mitigation
Source: PLoS One. 2018 Oct 31;13(10):e0204369. doi: 10.1371/journal.pone.0204369 (PMC6209147; doi:10.1371/journal.pone.0204369)
Supplement: S10 Fig — (Left) Distribution of subjects in clusters based on their average contribution per round. (Right) Cumulative distribution function based on their average contribution per round. (PDF) [file pone.0204369.s010.pdf]

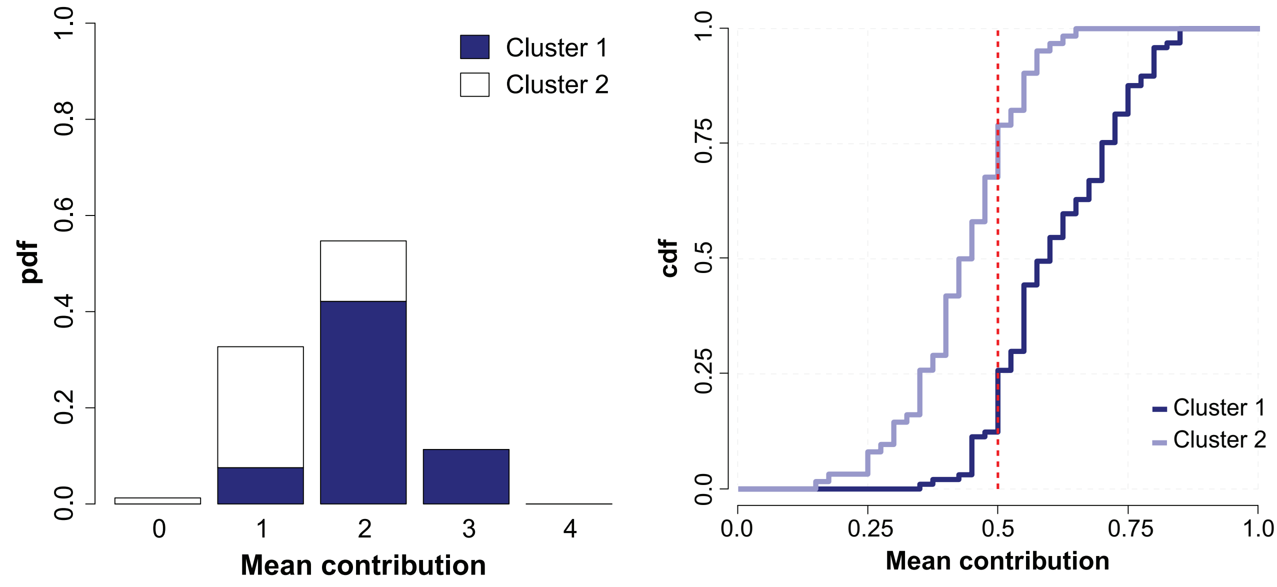

Fig S10: Equal Treatment Distributions. (Left) Distribution of subjects in clusters based on their average contribution per round. (Right) Cumulative distribution function based on their average contribution per round.
