# Supplementary material for: Resource heterogeneity leads to unjust effort distribution in climate change mitigation
Source: PLoS One. 2018 Oct 31;13(10):e0204369. doi: 10.1371/journal.pone.0204369 (PMC6209147; doi:10.1371/journal.pone.0204369)
Supplement: S11 Fig — (Top-Left) Optimal number of clusters. (Top-Right) Cluster consensus ratio. (Bottom) Item consensus ratio. (PDF) [file pone.0204369.s011.pdf]

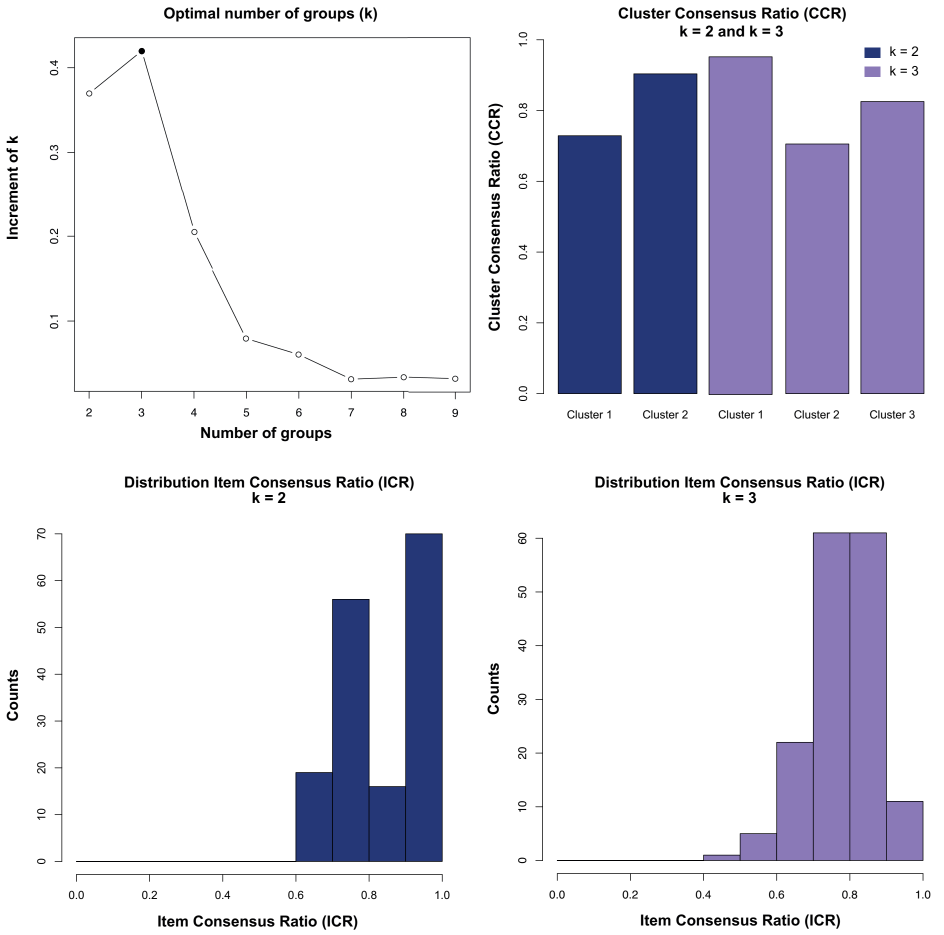

Fig S11: Unequal Treatment Clustering. (Top-Left) Optimal number of clusters. (Top-Right) Cluster consensus ratio. (Bottom) Item consensus ratio.
