# Supplementary material for: Resource heterogeneity leads to unjust effort distribution in climate change mitigation
Source: PLoS One. 2018 Oct 31;13(10):e0204369. doi: 10.1371/journal.pone.0204369 (PMC6209147; doi:10.1371/journal.pone.0204369)
Supplement: S13 Fig — First column displays the corresponding distribution of endowments at the beginning of the game. Second column pictures the hypothetical final distribution if all the participants contributed fairly, i.e. with half of their endowments. Finally in the third column, the observed final distribution of capitals is shown. The Gini coefficient is indicated in every case. (PDF) [file pone.0204369.s013.pdf]

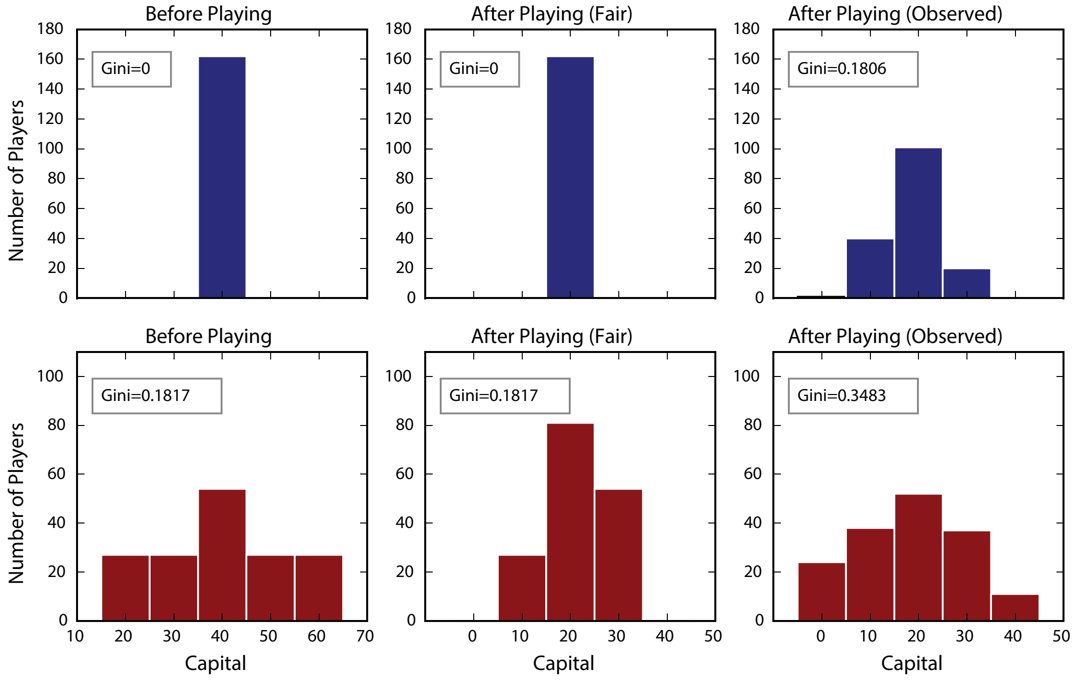

Fig S13: Distribution of capitals in different scenarios of the game for the equal treatment (first row in light blue) and the unequal treatment (bottom row in red). First column displays the corresponding distribution of endowments at the beginning of the game. Second column pictures the hypothetical final distribution if all the participants contributed fairly, i.e. with half of their endowments. Finally in the third column, the observed final distribution of capitals is shown. The Gini coefficient is indicated in every case.
