# Supplementary material for: Resource heterogeneity leads to unjust effort distribution in climate change mitigation
Source: PLoS One. 2018 Oct 31;13(10):e0204369. doi: 10.1371/journal.pone.0204369 (PMC6209147; doi:10.1371/journal.pone.0204369)
Supplement: S14 Fig — Average earnings and standard error of the mean (95% CI) regarding treatment and endowments. (PDF) [file pone.0204369.s014.pdf]

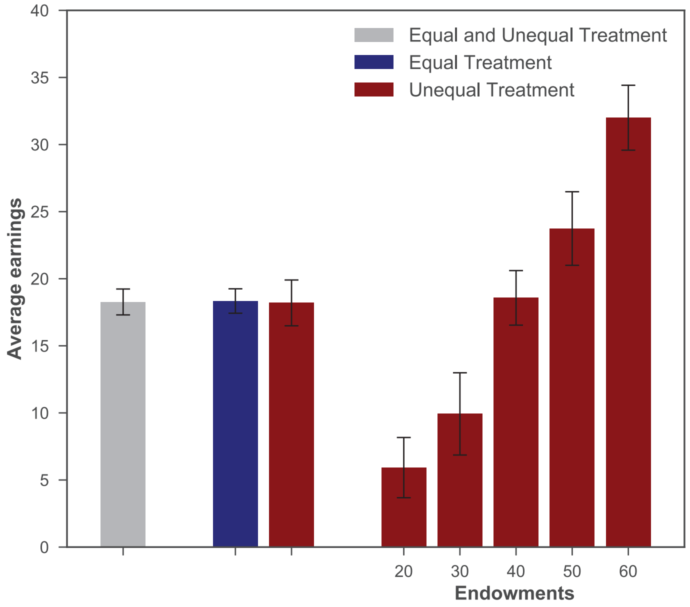

Fig S14: Earnings. Average earnings and standard error of the mean (95% CI) regarding treatment and endowments.
