# Supplementary material for: Resource heterogeneity leads to unjust effort distribution in climate change mitigation
Source: PLoS One. 2018 Oct 31;13(10):e0204369. doi: 10.1371/journal.pone.0204369 (PMC6209147; doi:10.1371/journal.pone.0204369)
Supplement: S15 Fig — (Left) Duration of a game, mean and standard error of the mean (95% CI), per treatment. (Right) Evolution of decision making times over round. (PDF) [file pone.0204369.s015.pdf]

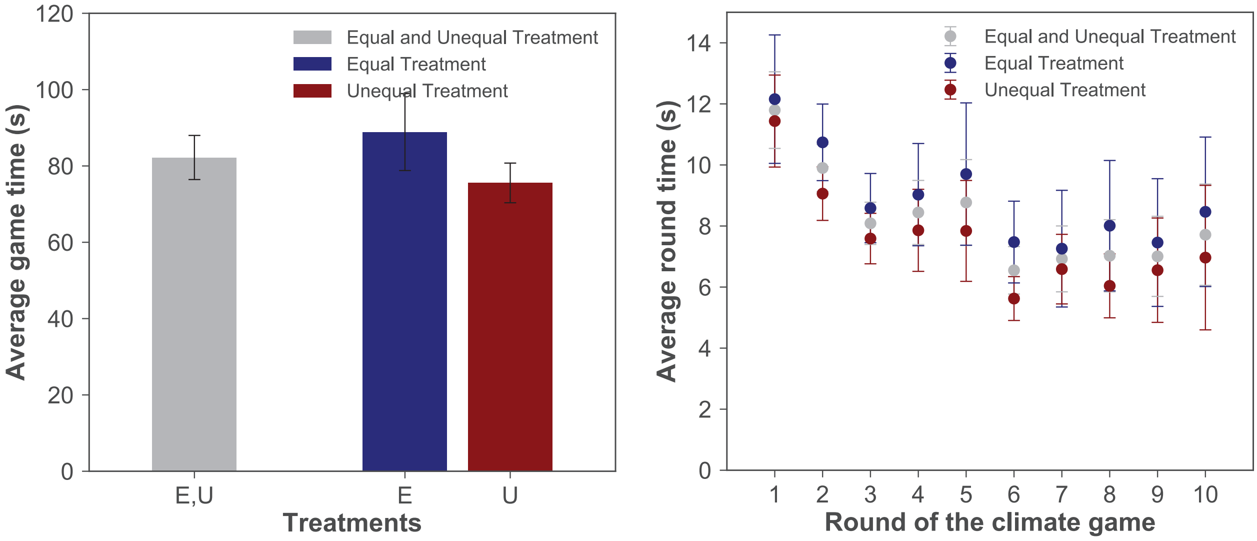

Fig S15: Decision making times. (Left) Duration of a game, mean and standard error of the mean (95% CI), per treatment. (Right) Evolution of decision making times over round.
