# Supplementary material for: Resource heterogeneity leads to unjust effort distribution in climate change mitigation
Source: PLoS One. 2018 Oct 31;13(10):e0204369. doi: 10.1371/journal.pone.0204369 (PMC6209147; doi:10.1371/journal.pone.0204369)
Supplement: S16 Fig — Images of the character created by Mensula Studio are licensed under CC BY 4.0. (PDF) [file pone.0204369.s016.pdf]

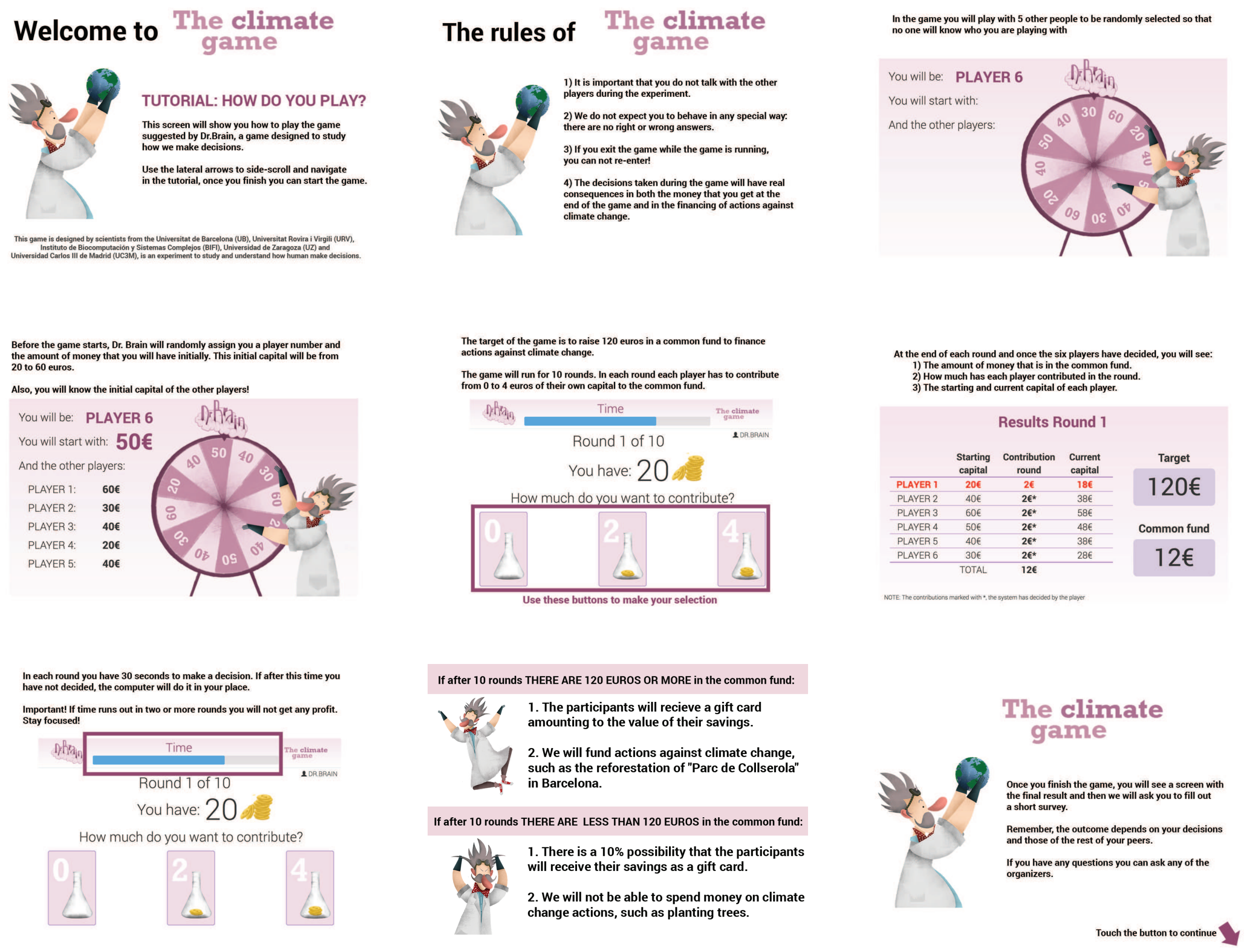

Fig S16: Screenshots of the tutorial shown before the experiment. Images of the character created by Mensula Studio are licensed under CC BY 4.0.
