# Supplementary material for: Resource heterogeneity leads to unjust effort distribution in climate change mitigation
Source: PLoS One. 2018 Oct 31;13(10):e0204369. doi: 10.1371/journal.pone.0204369 (PMC6209147; doi:10.1371/journal.pone.0204369)
Supplement: S1 Table — (PDF) [file pone.0204369.s017.pdf]

Table S1: Example of user's contribution normalization and binning in a particular game.

**Round**	**1**	**2**	**3**	**4**	**5**	**6**	**7**	**8**	**9**	**10**
Contribution[1]	4	3	4	3	2	2	4	3	3	0
Remaining to target[2]	120	102	82	61	49	26	21	8	1	-5
Common Fund[3]	0	18	38	59	71	84	99	112	119	125
**Binning (bin=24)[4]**	**0-23**		**24-47**	**48-71**		**72-95**	**96-119**			**$\geqslant$ 120**
Av. Cont.[5]	3.5		4	2.5		2	3.3			0
Av. Cont. Norm.[6]	0.875		1	0.625		0.5	0.83			0

[1] Contribution of a single user over the game (10-rounds). [2] Capital remaining to achieve the goal in a particular game (120€ at the beginning of the game). [3] Capital contributed and accumulated in each round of a particular game. [4] Binning the common fund in groups of 24. [5] Average contribution of a single user in the bin. [6] Average contribution normalized of a single user in the bin.
