# Supplementary material for: Resource heterogeneity leads to unjust effort distribution in climate change mitigation
Source: PLoS One. 2018 Oct 31;13(10):e0204369. doi: 10.1371/journal.pone.0204369 (PMC6209147; doi:10.1371/journal.pone.0204369)
Supplement: S2 Table — (PDF) [file pone.0204369.s018.pdf]

Table S2: Cohort analysis of game contributions in games with and without minors.

Treatment	Minor	Mean	sd	n	se	t	df	p-value
Both	Yes	129.7	10.2	29	1.9	0.8	44.6	0.45
	No	132.2	13.4	25	2.7			
Equal	Yes	127.1	5.7	15	1.5	1.3	13.2	0.22
	No	133.4	16.1	12	4.6			
Unequal	Yes	132.4	13.3	14	3.5	-0.3	24.6	0.776
	No	131.0	10.9	13	3.0			
