# Supplementary material for: Resource heterogeneity leads to unjust effort distribution in climate change mitigation
Source: PLoS One. 2018 Oct 31;13(10):e0204369. doi: 10.1371/journal.pone.0204369 (PMC6209147; doi:10.1371/journal.pone.0204369)
Supplement: S3 Table — (PDF) [file pone.0204369.s019.pdf]

Table S3: Cohort analysis of contribution per endowment in groups of minors and adults.

Endowment	Treatment	Minor	Mean	SD	n	SE	t	df	p-value
20	Unequal	Yes	17	2.6	3	1.5	-1.7	5	0.149
		No	13.7	5.8	23	1.2			
30	Unequal	Yes	23.2	7.3	4	3.6	-0.9	4.3	0.399
		No	19.5	7.8	23	1.6			
40	Unequal	Yes	22.6	7	10	2.2	-0.6	14.1	0.575
		No	21.1	7.6	44	1.1			
40	Equal	Yes	21.4	6.2	28	1.2	0.2	37.4	0.801
		No	21.7	5.7	131	0.5			
50	Unequal	Yes	27.2	9.7	4	4.8	-0.2	3.5	0.830
		No	26.1	6.6	23	1.4			
60	Unequal	Yes	25.5	3.9	4	1.9	1.2	6.3	0.256
		No	28.4	6.4	23	1.3			
