# Supplementary material for: Resource heterogeneity leads to unjust effort distribution in climate change mitigation
Source: PLoS One. 2018 Oct 31;13(10):e0204369. doi: 10.1371/journal.pone.0204369 (PMC6209147; doi:10.1371/journal.pone.0204369)
Supplement: S4 Table — (PDF) [file pone.0204369.s020.pdf]

Table S4: Payoff and payoff normalized by relative fairness.

			Payoff			Payoff Normalized		
**Endowment**	**Treatment**	**n**	**Mean**	**SD**	**SE**	**Mean**	**SD**	**SE**
20	Unequal	26	5.92	5.56	1.09	0.59	0.56	0.11
30	Unequal	27	9.93	7.75	1.49	0.66	0.52	0.10
40	Unequal	54	18.57	7.45	1.01	0.93	0.37	0.05
40	Equal	159	18.34	5.8	0.46	0.92	0.29	0.02
50	Unequal	27	23.74	6.93	1.33	0.95	0.28	0.05
60	Unequal	27	32	6.11	1.18	1.07	0.2	0.04
