# Supplementary material for: Resource heterogeneity leads to unjust effort distribution in climate change mitigation
Source: PLoS One. 2018 Oct 31;13(10):e0204369. doi: 10.1371/journal.pone.0204369 (PMC6209147; doi:10.1371/journal.pone.0204369)
Supplement: S5 Table — (PDF) [file pone.0204369.s021.pdf]

Table S5: Pairwise comparation of payoff normalized by relative fairness.

Endowment	20‡	30‡	40†	40‡	50‡	60‡
**20‡**	-	**-0.5 (0.640)**	-2.9** (0.007)	-2.8** (0.008)	-2.9** (0.005)	-4.1*** ($3 \cdot 10^{-4}$)
**30‡**	**0.5 (0.640)**	-	-2.5* (0.018)	-2.4* (0.021)	-2.5* (0.015)	-3.8** (0.001)
**40†**	2.9** (0.007)	2.5* (0.018)	-	**-0.2 (0.834)**	**-0.6 (0.578)**	-3.3** (0.002)
**40‡**	2.8** (0.008)	2.4* (0.021)	**0.2 (0.834)**	-	**-0.3 (0.777)**	-2.1* (0.034)
**50‡**	2.9** (0.005)	2.5* (0.015)	**0.6 (0.578)**	**0.3 (0.777)**	-	**-1.8 (0.083)**
**60‡**	4.1*** ($3 \cdot 10^{-4}$)	3.8** (0.001)	3.3** (0.002)	2.1* (0.034)	**1.8 (0.083)**	-

† Equal treatment and ‡ Unequal treatment. The table displays the values of Welch t-tests (p-value) used to compare the differences of payoff's means among the groups. *significant at 5%; **significant at 1%; *** significance less than 0.1%.
