# Supplementary material for: Resource heterogeneity leads to unjust effort distribution in climate change mitigation
Source: PLoS One. 2018 Oct 31;13(10):e0204369. doi: 10.1371/journal.pone.0204369 (PMC6209147; doi:10.1371/journal.pone.0204369)
Supplement: S1 File — In the S1 File (PDF) we present further details about the experiment, statistical tests, clustering analysis and additional information. (PDF) [file pone.0204369.s022.pdf]

Supplementary Information

Resource heterogeneity leads to unjust effort distribution in climate change mitigation

Julian Vicens[a,b,c], Nereida Bueno-Guerra[d], Mario Gutiérrez-Roig[b,e], Carlos Gracia-Lázaro[f,g], Jesús Gómez-Gardeñes[f,h], Josep Perelló[b,c], Angel Sánchez[f,g,i,j], Yamir Moreno[f,g,k,l], and Jordi Duch[a,m]

[a] Departament d'Enginyeria Informàtica i Matemàtiques, Universitat Rovira i Virgili, Tarragona, Spain

[b] Departament de Física de la Matèria Condensada, Universitat de Barcelona, Barcelona, Spain

[c] Institute of Complex Systems UBICS, Universitat de Barcelona, Barcelona, Spain

[d] Department of Psychology, Comillas Pontifical University, Madrid, Spain

[e] Behavioural Science Group, Warwick Business School, University of Warwick, Coventry, United Kingdom

[f] Institute for Biocomputation and Physics of Complex Systems (BIFI), University of Zaragoza, Zaragoza, Spain

[g] Unidad Mixta Interdisciplinar de Comportamiento y Complejidad Social (UMICCS), UC3M-UV-UZ, Spain

[h] Department of Condensed Matter Physics, University of Zaragoza, Zaragoza, Spain

[i] Grupo Interdisciplinar de Sistemas Complejos (GISC), Unidad de Matemática, Modelización y Ciencia Computacional, Universidad Carlos III de Madrid, Leganés, Spain

[j] Institute UC3M-BS of Financial Big Data, Universidad Carlos III de Madrid, Getafe, Spain

[k] Department of Theoretical Physics, University of Zaragoza, Zaragoza, Spain

[l] ISI Foundation, Turin, Italy

[m] Northwestern Institute on Complex Systems, Northwestern University, Evanston, USA

Contents

1 Supplementary Notes **3**

1.1 Experiment participation . . . 3

1.1.1 Demographics . . . 3

1.2 Selection strategy . . . 3

1.3 Game evolution . . . 4

1.3.1 Phases of the game . . . 4

1.3.2 Binning . . . 5

1.4 Individual Behavior . . . 5

1.4.1 Clustering Analysis . . . 5

1.4.2 Equal Treatment . . . 6

1.4.3 Unequal Treatment . . . 6

1.5 Capital Inequality . . . 6

1.6 Earnings . . . 7

1.7 Decision Making Times . . . 7

1.8 Questionnaire . . . 7

1.9 Tutorial . . . 8

1 Supplementary Notes

1.1 Experiment participation

The experiment was performed during the games festival (Festival de Jocs de Taula) DAU in Barcelona, in December 2015, over a period of two days.

We collected data from 324 participants who were randomly recruited by asking of DAU visitors if they would take part in our research. Each participant was given a tablet to play the game on. Before the actual experiment started, the subjects were passed a tutorial (see Sec. 9) with the game instructions, to understand the basics and mechanics of the games, the goal and the consequences of their contributions. Also, some of our team members were checking that the participants understood the instructions during the tutorial period (but not during the actual game). Once the participants had understood it, they pressed the button to indicate the system that they were ready to start playing. Once a group of six participants was ready, we started the game. We performed 54 games and collected 3240 actions.

As we explain in the tutorial the participants had a limited time to take their decisions (30 seconds), after this time if they didn't decide, the computer did it in their place. 320 subjects contributed valid data so 3200 valid decisions in 50 games.

1.1.1 Demographics

The average age among our valid participants was 32.15 (SD=13.04), with a proportion of 41.88% females and 58.12% males (see Fig S1).

Effect of gender by contribution

The average (SD) contribution by gender is: 2.24 € (0.68) in females and 2.13 € (0.72) in males. There are no significant differences in the means by gender (t-test, t: -1.35 p: 0.18).

Effect of minors

We consider minors the participants with an age younger than 18. The differences between the behaviour of participants minors and adults, and their influence in the games is described with two parameters: (i) The amount contributed in the common fund in games with minors and adults (29) and games with only adults (25) is 129.7 € (10.2) and 132.2 € (13.4) respectively, no significant differences of the means (t-test, t: 0.8 p: 0.45) (see Table S2); (ii) in the same line, the individual contribution do not show significant differences for any of the endowment (see Table S3).

1.2 Selection strategy

We also studied the effect of including more decision options to the participants, instead of allowing to invest 0 €, 2 € or 4 € (control setting) the participants could select to invest between 0 € and 4 € (intervention setting). Offering more options to invest allows the existence of more complex investing

strategies, since the participants can tune more accurately how much they want to contribute in each round (see Fig S2).

The capital of participants decreased over the game in a different way depending on their endowments. In the unequal treatment, the capital of participants with low endowments decreased faster than the wealthy subjects, partly because the effort to contribute is greater for the poor participants and every single contribution reduced substantially their capital in comparison with the others participants.

The proportion of capital saved in both treatments have no significant difference (t-test $p < 0.05$) if we compare participants with same endowments (see Fig S3(left)). However we observe in the evolution of the average capital grouped by endowments how adding more options creates a significantly larger gap between the average contribution of richer (50/60 €) and poorer participants (20/30 €). It is also interesting to observe that while the gap is created in the first five rounds, it keeps getting larger until the end of the game (see Fig S3(right)).

1.3 Game evolution

In our experiment we collected how much one subject contributed in each round (Fig S4). In order to study and infer the existence of behavioral groups or patterns in the data, we first normalized the data so as to find the best classification.

First of all, we normalized the contribution done in each round based on the initial endowments. This is needed in order to compare meaningfully contributions of subjects in the unequal treatment with low endowments (20/30 €), mid endowments (40 €), and high endowments (50/60 €), and all of them with the subjects of the equal treatment where all subjects have the same endowment (40 €). Note that with this procedure, a normalized contribution of 0.5 represents the fair contribution in all treatments and all endowments.

By looking at the evolution of the game, we observe that each group reaches the target in different rounds −between the 6th and the 10th round (see Fig S5). Therefore, to make the contributions comparable we decided to analyze the evolution of the game not according to the contributions per round, but instead by calculating the average contribution in regard to the accumulated capital in the pot at the beginning of the round.

1.3.1 Phases of the game

The analysis of the evolution of the game according to the amount contributed to the common pot provides a better description of the behavior of the groups, and allows distinguishing different phases of the game. For instance (see Fig S6), we have observed that at the beginning of the game the subjects contribute a lot and explore the contributions of the other participants. However, when they get closer to the goal, there is an important change on how people contribute: their contributions decrease when they see that the goal is close to being achieved (see Fig S7).

1.3.2 Binning

To study the phases of the game, that is, the evolution independently of the rounds, we bin the rounds according to the accumulated capital in the common fund, and calculate the participants's average contribution per bin and normalize it (see Table S1 for an example on how this normalization is done).

1.4 Individual Behavior

To study the contribution strategies of the participants of our experiment we first checked whether they follow ”pure” strategies by looking at the total of their contributions over the whole game. Note that we only take into account the contributions before the target was reached, and also that the strategies are conditioned by the inequality of endowments. We consider three ”pure” strategies: (i) free-riders, those with a contribution of 0 €; (ii) fairers, those that have an average contribution per round of 1 € (20 €), 1.5 € (30 €), 2 € (40 €) and so on; and (iii) altruists, those who contribute the maximum that it is possible according to their initial capital, with average round contributions of 2 €, 3 €, 4 €, 4 € and 4 € per endowments of 20 €, 30 €, 40 €, 50 € and 60 € respectively (see Fig S8). We observe that the subjects rarely follow those ’pure’ strategies, only 42 out of 320 subjects followed a particular strategy: 1 free-rider, 30 fairers and 11 altruists.

In light of the above results and the difficulties to find clear strategies in the experiment, we next ran an unsupervised algorithm to understand the variety of subject's behavior over the game.

1.4.1 Clustering Analysis

We hypothesized that there exist different strategies of cooperation in our dataset that can not be described in terms of pure strategies due to their complexity, but that they could be revealed using unsupervised learning techniques. Hence, we ran hierarchical cluster algorithm on our data to analyze the structure of subject's contributions. We represent the sequence of contribution as explained above (see Table S1), creating a matrix of contribution, where every row represents a vector of individual contributions over the game. We use the accumulated capital in the common fund instead of rounds because every game could finish in a different round (see Fig S5), and a bin = 24 due to the rapid evolution of the game. Therefore, in each cell, we have the average contribution normalized (regarding the endowment) in the round that grouped every bin.

We implemented an agglomerative hierarchical clustering strategy (using the hclust package in R) to find an initial approximation of groups using ward.D2 and euclidean distances. The agglomerative strategies consist of a ”bottom-up” approach, each user contribution starts in its own cluster and pairs of clusters are merged in each step based on the optimal value of an error sum of squares (Ward's method). With the objective to determine the number of groups that better fits with our data, we ran an algorithm (NBCluster package in R) that computes 26 indexes and recommends the optimal number of clusters according to the majority rule.

Once we know that there is sufficient evidence to find groups of participants with different strategies and behavior, and to ensure that the clustering results are robust and reliable we ran an implementation of consensus clustering (ConsensusClusteringPlus package in R). Consensus clustering determines the

number of clusters and computes consensus values such as item consensus and cluster consensus. Item consensus represents the membership of an item with all items in a particular cluster, this value indicates if a particular item is a pure member of the cluster or if it is unstable. Cluster consensus provides information about the consensus between members of a group, high values indicate high stability.

The parameters we used to perform the calculation illustrated in the next sections are: maximum evaluated k of 9 so that groups count of 2 to 9 are evaluated; 1000 re-samplings, agglomerative hierarchical clustering algorithm, euclidean distances and ward.D2 linkage.

1.4.2 Equal Treatment

The equal treatment of the experiment includes 162 subjects (27 games), of which 159 subjects contributed with valid actions. In order to analyze their individual strategies we created a matrix with the average contribution in each stage of the game binned by the accumulated capital in the common fund, and then we computed the cluster consensus in our equal treatment dataset.

The number of clusters that better fits with our data and maximizes the consensus values is 2 (see Fig S9). The average (SD) consensus clustering ration for 2 groups is 0.75 (0) and the average of item consensus is 0.75 (0.08). Cluster 1 is composed by 97 subjects (61%) while cluster 2 is formed by 62 subjects (39%). Fig S10 represents the distribution of subjects (pdf) and the cumulative distribution function (cdf) of their average contribution in both clusters.

1.4.3 Unequal Treatment

The unequal treatment includes 162 subjects (27 games), of which 161 contributed valid data. The matrix of contributions was formed in the same way that in the equal case.

The consensus cluster approach concluded that 3 clusters is the most stable number of groups in this treatment, see Fig S11. In this case, the average (SD) consensus clustering ratio is 0.83 (0.12) and the average of item consensus ratio is 0.78 (0.09).

In contrast to the equal treatment, in the unequal scenario a new group appears. The composition of clusters is: cluster 1, 12 subjects (7.45%); cluster 2, 70 subjects (43.48%); and cluster 3, 79 subjects (49.07%). The most populated cluster is the one composed by subjects that contributed around and below the fair contribution. Fig S12 represents the distribution of subjects (pdf) and the cumulative distribution function (cdf) of their average contribution in both clusters.

1.5 Capital Inequality

As shown throughout the main text, participants with less initial capital tend to contribute much more in relative terms. As a result of this behavior, we also observe an increase of inequality that we would not observe if the participants contributed the amount defined as fair in the main text (see Fig S13). We also measure such inequality by means of the Gini Coefficient, a standard measure in economics used extensively for studying income distributions and its inequality.

1.6 Earnings

Once the game finished and if the subjects achieved the goal, they kept the capital not contributed and received it in the form of a gift card. The average (standard deviation) earnings among all the subjects were 18.21 € (8.8). There exist no significant differences in the means (t-test; t:0.26, p:0.8) between the equal treatment 18.33 € (5.8) and the unequal treatment 18.08 € (11). Fig S14 illustrates earnings regarding of endowments and treatments in detail.

1.7 Decision Making Times

The platform designed to carry out the experiment also records the decision making times and the duration of a round, and we can also obtain the total playing time by suming the durations of rounds in a game. The average (standard deviation) duration of all games was 82.21 s (20.98), and the average (standard deviation) duration of a round was 8.22 s (4.47). There are significant differences in the mean (t-test; t:2.41, p:0.019) between equal treatment 88.88 s (25.01) and unequal treatment 75.55 s (12.89). The decision times decrease as rounds go on, especially in the first five rounds, and subsequently stabilizes (see Fig S15).

1.8 Questionnaire

After the collective-risk dilemma ended, as a last stage of the experiment, for each group of subjects we asked a questionnaire with two sets of questions: (i) how they made decisions in the game and (ii) some basic concepts about climate change. The questionnaire was also presented identically in three languages (Catalan, Spanish and English), here we present the questions in English.

Question 1: *Had you previously participated in Citizen Science experiments?* Answers: a.*No*; b.*Yes*; c.*Yes, in previous DAU experiments.*

Question 2: *Did you like the experience?* Answers: a.*Very much*; b.*Somewhat*; c.*Not really*; d.*Not at all.*

Question 3: *At the beginning of the experiment, did you expect to reach the 120 € target?* Answers: a.*Yes, from the beginning*; b.*Yes, after a few rounds*; c.*No, from the beginning*; d.*No, after a few rounds.*

Question 4: *Generally, if others are contributing little, I should also contribute little.* Answers: a.*Agree*; b.*Disagree*; c.*My contribution should not depend on this*; d.*n/a.*

Question 5: *Generally, if others are contributing a lot, I should also contribute a lot.* Answers: a.*Agree*; b.*Disagree*; c.*My contribution should not depend on this*; d.*n/a.*

Question 6: *I think there have been players who have taken advantage of the generosity of others to maintain their capital.* Answers: a.*Agree*; b.*Disagree*; c.*n/a.*

Question 7: *The contributions of each player should be proportional to their capital: those who have started with more should contribute more and those who have started with less should contribute less.* Answers: a.*Agree*; b.*Disagree*; c.*n/a.*

Question 8: *The contributions of each player should be fair, because the benefits of the common fund affect everyone equally, as well as the risk of losing everything.* Answers: a.*Agree*; b.*Disagree*; c.*n/a.*

Question 9: *It seems fair to me that a player with a lot of capital should get money at the end, if he has contributed at least half of what he had.* Answers: a.*Agree*; b.*Disagree*; c.*n/a*.

Question 10: *When the polluting gases prevent the rays of the Sun from coming out of the Earth, it is due to...* Answers: a.*Doppler Effect*; b.*Greenhouse Effect*; c.*Faraday Effect*; d.*Refrigerator Effect.*

Question 11: *Which of the following countries is the most polluting in the world?* Answers: a.*U.S*; b.*Italy*; c.*China*; d.*Japan.*

Question 12: *Which of the following elements is the least polluting?* Answers: a.*Oil*; b.*Carbon*; c.*Solar energy*; d.*Nuclear energy.*

Question 13: *Which international treaty tries to regulate CO2 emissions to the atmosphere?* Answers: a.*Declaration of Helsinki*; b.*Kyoto Protocol*; c.*Schengen Agreement*; d.*Treaty of Versailles.*

Question 14: *What is the total number of gaseous pollutants emitted by each individual?* Answers: a.*Carbon footprint*; b.*Eco-Impact*; c.*Individual gas fee*; d.*Reduced environmental cost.*

Question 15: *According to the economist Nicolas Stern, if urgent measures are not implemented, what costs could climate change represent in 2050, as a percentage of world GDP?* Answers: a.*2%*; b.*5%*; c.*15%*; d.*20%*

1.9 Tutorial

Before the collective-risk dilemma started, we showed to each group of subjects a tutorial that was included in the same platform used to participate in the game. The subjects were assisted by researchers who answered any questions that came up about the experiment. The tutorial was presented identically in three languages (Catalan, Spanish and English). Here we show the tutorial screens and text of the English version.

Tutorial Screen 1 (Fig S16a). *Welcome to The Climate Game. TUTORIAL: HOW DO YOU PLAY?. This screen will show you how to play the game suggested by Dr.Brain, a game designed to study how we make decisions. This game is designed by scientists from the Universitat de Barcelona (UB), Universitat Rovira i Virgili (URV), Instituto de BiocomputaciÃşn y Sistemas Complejos (BIFI), Universidad de Zaragoza (UZ) and Universidad Carlos III de Madrid (UC3M), is an experiment to study and understand how human make decisions. Use the lateral arrows to side-scroll and navigate in the tutorial, once you finish you can start the game.*

Tutorial Screen 2 (Fig S16b). *The rules of The Climate Game. 1) It is important that you do not talk with the other players during the experiment. 2) We do not expect you to behave in any special way: there are no right or wrong answers. 3) If you exit the game while the game is running, you can not re-enter! 4) The decisions taken during the game will have real consequences in both the money that you get at the end of the game and in the financing of actions against climate change.*

Tutorial Screen 3 (Fig S16c). *In the game you will play with 5 other people to be randomly selected so that no one will know who you are playing with*

Tutorial Screen 4 (Fig S16d). *Before the game starts, Dr. Brain will randomly assign you a player number and the amount of money that you will have initially. This initial capital will be from 20 to 60 euros. Also, you will know the initial capital of the other players!*

Tutorial Screen 5 (Fig S16e).*The target of the game is to raise 120 euros in a common fund to finance actions against climate change. The game will run for 10 rounds. In each round each player has to contribute from 0 to 4 euros of their own capital to the common fund.*

Tutorial Screen 6 (Fig S16f).*At the end of each round and once the six players have decided, you will see: 1) The amount of money that is in the common fund. 2) How much has each player contributed in the round. 3) The starting and current capital of each player.*

Tutorial Screen 7 (Fig S16g).*In each round you have 30 seconds to make a decision. If after this time you have not decided, the computer will do it in your place. Important! If time runs out in two or more rounds you will not get any profit. Stay focused!*

Tutorial Screen 8 (Fig S16h).*If after 10 rounds THERE ARE 120 EUROS OR MORE in the common fund: 1. The participants will recieve a gift card amounting to the value of their savings. 2. We will fund actions against climate change, such as the reforestation of "Parc de Collserola" in Barcelona. If after 10 rounds THERE ARE LESS THAN 120 EUROS in the common fund: 1. There is a 10% possibility that the participants will receive their savings as a gift card. 2. We will not be able to spend money on climate change actions, such as planting trees.*

Tutorial Screen 9 (Fig S16i).*The Climate Game. Once you finish the game, you will see a screen with the final result and then we will ask you to fill out a short survey. Remember, the outcome depends on your decisions and those of the rest of your peers. If you have any questions you can ask any of the organizers. Touch the button to continue*
